# Excelentes Resultados Hospitalares em TAVI Bicúspide. Mas e Agora?

**DOI:** 10.36660/abc.20260398

**Published:** 2026-07-15

**Authors:** Maria Cristina Meira Ferreira

**Affiliations:** 1 Universidade Federal do Rio de Janeiro Rio de Janeiro RJ Brasil Universidade Federal do Rio de Janeiro, Rio de Janeiro, RJ – Brasil

**Keywords:** Substituição da Valva Aórtica Transcateter, Doença da Valva Aórtica Bicúspide, Estenose da Valva Aórtica

O implante transcateter de valva aórtica (TAVI) se expandiu rapidamente na última década, impulsionado por ensaios clínicos randomizados, registros contemporâneos e a evolução progressiva das recomendações das diretrizes internacionais.^1^ Paralelamente, estudos multicêntricos têm demonstrado cada vez mais o uso de TAVI em populações mais complexas e com menor risco cirúrgico, incluindo pacientes com anatomia bicúspide.^2^ À medida que a experiência acumulada aumentou e novas gerações de valvas transcateter foram incorporadas à prática clínica, cenários antes considerados desafiadores começaram a ser explorados com mais frequência, incluindo a estenose aórtica associada à valva aórtica bicúspide.

No entanto, permanecem dúvidas sobre até que ponto as evidências derivadas da estenose aórtica tricúspide podem ser extrapoladas diretamente para pacientes com anatomia bicúspide. Como destacado por Hirji et al.,^3^ a doença da valva bicúspide representa uma condição fundamentalmente distinta da estenose tricúspide degenerativa, caracterizada por calcificação assimétrica, geometria anular elíptica e alta prevalência de aortopatia associada. Essas características introduzem desafios capazes de afetar tanto o sucesso imediato do procedimento quanto o desempenho e a durabilidade da valva a longo prazo.

Revisões contemporâneas reforçam que a doença da valva bicúspide não deve ser interpretada como uma única entidade anatômica, mas sim como um espectro heterogêneo de apresentações morfológicas com distintas implicações técnicas e prognósticas.^4-6^ Diferentes fenótipos, particularmente de acordo com a classificação de Sievers, exibem padrões variáveis de fusão das cúspides, distribuição de calcificação e anatomia da raiz da aorta, influenciando diretamente o dimensionamento, a expansão da prótese, a regurgitação paravalvar e a durabilidade do dispositivo. Estudos recentes sugerem que subtipos anatômicos específicos podem demonstrar distintos comportamentos no procedimento e na evolução clínica após TAVI.^7-9^ Nesse contexto, a ausência de uma caracterização anatômica detalhada da morfologia da valva bicúspide no presente estudo limita a interpretação dos resultados observados, uma vez que a falta de definição do fenótipo impossibilita determinar se subgrupos potencialmente mais complexos foram adequadamente representados na análise.^10^

Apesar dessas limitações anatômicas e metodológicas, os excelentes resultados intra-hospitalares relatados pelos autores reforçam a crescente viabilidade do procedimento de TAVI em pacientes com valva aórtica bicúspide, particularmente em centros experientes com seleção anatômica adequada. A baixa mortalidade e o perfil favorável de complicações imediatas refletem a evolução contemporânea da técnica do procedimento, do planejamento baseado em imagem e da tecnologia de valvas transcateter.^10^ No entanto, os resultados intra-hospitalares representam apenas uma dimensão do sucesso terapêutico nessa população. Embora a ausência de eventos precoces seja encorajadora, ela não se traduz necessariamente em equivalência clínica a médio e longo prazo.^3,11^ Essa discussão se torna particularmente relevante porque pacientes com doença valvar bicúspide frequentemente desenvolvem estenose aórtica em idades mais jovens, aumentando a importância da durabilidade da valva, do desempenho hemodinâmico a longo prazo e da potencial necessidade de intervenções futuras ao longo da vida.^11^

Além da heterogeneidade anatômica, TAVI em anatomia bicúspide permanece associado a desafios técnicos específicos relacionados à passagem da valva, ao dimensionamento da prótese, à expansão assimétrica da valva e à interação entre o dispositivo e a anatomia supra-anular.^6^ Evidências recentes demonstram que a calcificação extensa do rafe e certas configurações anatômicas podem influenciar diretamente a regurgitação paravalvar, os gradientes residuais e a falha do dispositivo após TAVI.^9^ Diferenças entre as plataformas de valvas transcateter também podem afetar os resultados do procedimento, incluindo a necessidade de implante de uma segunda valva e complicações anulares.^12^ Esses achados reforçam que TAVI em pacientes bicúspides depende não apenas da viabilidade do procedimento, mas também de um planejamento altamente individualizado.

Outro aspecto relevante é que uma proporção substancial das evidências disponíveis deriva predominantemente de registros observacionais e análises retrospectivas.^3,4^ Muitos estudos excluem pacientes com anatomias bicúspides mais complexas, limitando a generalização dos resultados.^4,6^ Como enfatizado por Hirji et al.,^3^ existe atualmente uma possível discrepância entre o entusiasmo tecnológico a cerca de TAVI e a maturidade das evidências disponíveis para pacientes bicúspides mais jovens. Além disso, a duração do acompanhamento permanece relativamente curta, limitando conclusões robustas sobre a durabilidade da prótese, a deterioração estrutural da valva e a necessidade de reintervenção. A ausência de avaliação ecocardiográfica pós-alta no presente estudo também limita interpretações mais amplas sobre o sucesso do procedimento. Em pacientes com anatomia bicúspide, o acompanhamento longitudinal torna-se particularmente importante para avaliar não apenas o sucesso técnico imediato, mas também o desempenho funcional e a durabilidade da valva a longo prazo. Nesse contexto, dados recentes derivados do NOTION-2 sugerem que, embora os resultados iniciais sejam encorajadores, ainda persistem incertezas importantes sobre a evolução clínica a longo prazo em pacientes bicuspids.^11^

Assim, os resultados apresentados pelos autores devem ser interpretados como uma importante demonstração da viabilidade contemporânea do procedimento de TAVI em anatomia bicúspide, mas não necessariamente como evidência definitiva de benefício sustentado a longo prazo. Embora derivados de uma experiência ainda limitada em número de pacientes, esses achados são particularmente relevantes por representarem a experiência nacional contemporânea em um cenário ainda pouco explorado no contexto brasileiro. Em um país marcado por significativas disparidades regionais no acesso e na incorporação de novas tecnologias, a descrição de desfechos locais contribui substancialmente para a compreensão da aplicabilidade dessa estratégia terapêutica na prática clínica de pacientes com anatomia bicúspide.
