# Supplementary material for: Excelentes Resultados Hospitalares em TAVI Bicúspide. Mas e Agora?
Source: Arq Bras Cardiol. 2026 Jul 15;123(6):e20260398. [Article in Portuguese] doi: 10.36660/abc.20260398 (PMC13399414; doi:10.36660/abc.20260398)
Supplement: Translation

# Translation: Excellent In-Hospital Outcomes in Bicuspid TAVI. But What Comes Next?

**DOI:** 10.36660/abc.20260398i

**Authors:** Maria Cristina Meira Ferreira

**Affiliations:** 1 Rio de Janeiro RJ Brazil Universidade Federal do Rio de Janeiro, Rio de Janeiro, RJ – Brazil

**Keywords:** Transcatheter Aortic Valve Replacement, Bicuspid Aortic Valve Disease, Aortic Valve Stenosis

Transcatheter aortic valve implantation (TAVI) has expanded rapidly over the last decade, driven by randomized trials, contemporary registries, and the progressive evolution of international guideline recommendations.^1^ In parallel, multicenter studies have increasingly demonstrated the use of TAVI in more complex and lower surgical risk populations, including patients with bicuspid anatomy.^2^ As accumulated experience increased and newer generations of transcatheter valves were incorporated into clinical practice, scenarios previously considered challenging began to be explored more frequently, including aortic stenosis associated with a bicuspid aortic valve.

However, questions remain regarding the extent to which evidence derived from tricuspid aortic stenosis can be directly extrapolated to patients with bicuspid anatomy. As highlighted by Hirji et al.,^3^ bicuspid valve disease represents a fundamentally distinct condition from degenerative tricuspid stenosis, characterized by asymmetric calcification, elliptical annular geometry, and a high prevalence of associated aortopathy. These characteristics introduce challenges capable of affecting both immediate procedural success and long-term valve performance and durability.

Contemporary reviews reinforce that bicuspid valve disease should not be interpreted as a single anatomical entity, but rather as a heterogeneous spectrum of morphological presentations with distinct technical and prognostic implications.^4-6^ Different phenotypes, particularly according to the Sievers classification, exhibit varying patterns of cusp fusion, calcification distribution, and aortic root anatomy, directly influencing sizing, prosthesis expansion, paravalvular leak, and device durability. Recent studies suggest that specific anatomical subtypes may demonstrate distinct procedural behavior and clinical evolution after TAVI.^7-9^ In this context, the absence of detailed anatomical characterization of bicuspid valve morphology in the presented study limits interpretation of the observed results, since the lack of phenotype definition makes it impossible to determine whether potentially more complex subgroups were adequately represented in the analysis.^10^

Despite these anatomical and methodological limitations, the excellent in-hospital outcomes reported by the authors reinforce the growing feasibility of TAVI in patients with a bicuspid aortic valve, particularly in experienced centers with appropriate anatomical selection. The low mortality and favorable profile of immediate complications reflect the contemporary evolution of procedural technique, imaging-based planning, and transcatheter valve technology.^10^ Nevertheless, in-hospital outcomes represent only one dimension of therapeutic success in this population. Although the absence of early events is encouraging, it does not necessarily translate into clinical equivalence in the mid- and long-term.^3,11^ This discussion becomes particularly relevant because patients with bicuspid valve disease frequently develop aortic stenosis at younger ages, increasing the importance of valve durability, long-term hemodynamic performance, and the potential need for future interventions throughout life.^11^

Beyond anatomical heterogeneity, TAVI in bicuspid anatomy remains associated with specific technical challenges related to valve crossing, prosthesis sizing, asymmetric valve expansion, and the interaction between the device and supra-annular anatomy.^6^ Recent evidence demonstrates that extensive raphe calcification and certain anatomical configurations may directly influence paravalvular leak, residual gradients, and device failure following TAVI.^9^ Differences between transcatheter valve platforms may also affect procedural outcomes, including the need for second-valve implantation and annular complications.^12^ These findings reinforce that TAVI performance in bicuspid patients depends not only on procedural feasibility but also on highly individualized planning.

Another relevant aspect is that a substantial proportion of the available evidence is predominantly derived from observational registries and retrospective analyses.^3,4^ Many studies exclude patients with more complex bicuspid anatomies, limiting the generalizability of the results.^4,6^ As emphasized by Hirji et al.,^3^ there is currently a potential mismatch between the technological enthusiasm surrounding TAVI and the maturity of the evidence available for younger bicuspid patients. In addition, follow-up duration remains relatively short, limiting robust conclusions regarding prosthetic durability, structural valve deterioration, and the need for reintervention. The absence of post-discharge echocardiographic evaluation in the presented study also limits broader interpretations regarding procedural success. In patients with bicuspid anatomy, longitudinal follow-up becomes particularly important to assess not only immediate technical success, but also long-term functional valve performance and durability. In this context, recent data derived from NOTION-2 suggest that although early outcomes are encouraging, important uncertainties regarding long-term clinical evolution in bicuspid patients still persist.^11^

Thus, the results presented by the authors should be interpreted as an important demonstration of the contemporary feasibility of TAVI in bicuspid anatomy, but not necessarily as definitive evidence of sustained long-term benefit. Although derived from an experience still limited in patient numbers, these findings hold particular relevance for representing contemporary national experience in a scenario that remains underexplored in the Brazilian context. In a country marked by significant regional disparities in access to and incorporation of new technologies, the description of local outcomes contributes substantially to understanding the real-world applicability of TAVI in bicuspid anatomy.
